# Commentary: The Relationship of Bilingualism Compared to Monolingualism to the Risk of Cognitive Decline or Dementia: A Systematic Review and Meta-Analysis

**DOI:** 10.3389/fnagi.2017.00344

**Published:** 2017-10-25

**Authors:** John G. Grundy, John A. E. Anderson

**Affiliations:** Department of Psychology, York University, Toronto, ON, Canada

**Keywords:** bilingualism, dementia, meta-analysis, cognitive reserve, Alzheimer disease

Evidence suggests that lifelong bilingualism reshapes the brain and helps to prevent cognitive decline in older age (e.g., Bialystok, 2017). For example, Klein et al. (2016) showed bilingual countries have lower incidence rates of dementia than monolingual countries. Mukadam et al. (2017) conducted a meta-analysis examining the strength of the protective effect of bilingualism on dementia. The authors claim retrospective studies are often confounded by extraneous variables, whereas prospective studies are less susceptible. They conducted a meta-analysis and concluded that bilingualism does not protect from dementia given that only retrospective studies showed positive effects of bilingualism. However, three factors undermine their analysis and challenge their conclusions.

## Statistical issues

Although the authors state that 13 articles were included in their meta-analysis, the analysis included only *four* studies (Sanders et al., 2012; Yeung et al., 2014; Zahodne et al., 2014; Lawton et al., 2015), all of which were prospective. Thus, any conclusions comparing retrospective and prospective studies are not supported by statistical evidence. Nonetheless, of the four prospective studies included in the analysis, three included information regarding age at onset, the same measure as the retrospective studies. However, the authors did not analyze these data, arguing that “[t]hese outcomes were too heterogeneous to be combined in a meta-analysis.” We respectfully disagree given that meta-analyses are used precisely for the purpose of combining heterogeneous data and examining overall patterns. A more direct comparison of prospective and retrospective studies would be to include both types of studies within the same model using age at onset of dementia. It would have also been possible to include age at onset and incidence rate in a single analysis. One of the key features of a meta-analysis is that the individual studies included do not need to have the same dependent variables (Field and Gillett, 2010). The dependent variables are converted to effect sizes (e.g. Cohen's *d*), and it allows comparison of disparate outcomes (i.e., incidence/age of onset). Thus, including retrospective and prospective studies in a single meta-analysis would have allowed for the examination of whether the type of study (retrospective vs. prospective) moderated the overall effect. It is possible that such an analysis based on both study types would favor bilinguals and that the moderator analysis would not reveal a difference between the sources, but the analyses that are presented simply allow no conclusions.

## Conceptual issues

Retrospective and prospective studies have not examined the same outcome variable. Retrospective studies have examined *age at onset* of dementia symptoms, whereas prospective studies have examined dementia *incidence* rate. Given that prospective and retrospective studies have not studied the same outcome variable, this likely contributes to bilingualism being protective in one case and not the other—particularly when this relationship is not explicitly modeled with moderator analyses. Retrospective dementia studies often interpret their data according to a cognitive reserve perspective - the individual can cope with more neural degeneration (e.g., Perani et al., 2017). From this standpoint, one should not necessarily expect incidence rates of dementia to differ between monolinguals and bilinguals. Both groups will get the disease (eventually), but bilinguals should be able to withstand more disease pathology than monolinguals before reaching a critical drop-off point (hence the later age of onset for bilinguals). In other words, what is critical is not whether the individual gets the disease, but how quickly he or she accrues symptoms.

## Methodological issues

The authors' claim that retrospective studies are often confounded by extraneous variables to which prospective studies are less susceptible. While this is generally true, methodological flaws are pervasive in the prospective studies included in the analysis outweighing the benefits of the within-subject design. For example, Sanders et al. (2012) failed to ask their “monolinguals” if they spoke or understood other languages, a failing noted by Mukadam et al. Relatedly, Zahodne et al. (2014) failed to report second language proficiency and use and included “bilinguals” that reported speaking their second language “not well”. These limitations alone make it difficult to rule out the possibility that some of the monolinguals in these studies were, in fact, receptive bilinguals, and that some bilinguals were not very proficient in their second languages. It is therefore not entirely surprising that there were no significant differences in these cases. In contrast, many of the retrospective studies carefully controlled for multiple confounding variables, and these studies generally reported positive effects for bilingualism. For example, Craik et al. (2010) controlled for education and gender, as well as cognitive and occupational levels and found that bilinguals showed symptoms of dementia 5.1 years later than monolinguals. Alladi et al. (2013) examined a sample of individuals from an Indian population and revealed a delay of 4.5 years for bilinguals, independent of immigration status and the aforementioned controls.

A second methodological issue relates to Mukadam et al.'s use of the Newcastle-Ottawa scale (Wells et al., 2012). Controlling for quality is standard practice with meta-analyses, but the quality ratings were not consistent. Prospective studies automatically received extra points, increasing their likelihood of inclusion and therefore biasing the results. Retrospective studies were eliminated and in our view, were not fairly considered. Moreover, the interpretation of the quality scores was also inconsistent: the authors claim that a study by Bak et al. (2014), was of “lower quality,” yet two of the four studies included in the prospective meta-analysis received the same score.

## Conclusions

Well-controlled prospective studies would be informative and help uncover the differences between bilingual and monolingual individuals as they progress into Alzheimer's disease. These studies could also help to reveal the mechanisms allowing bilinguals to stave off symptoms for longer. However, such well-controlled prospective studies are still lacking. Of the studies that exist and provide adequate control for confounds, most of which are retrospective, bilingualism is generally found to be protective and delays symptoms for about 4.5 years. The overall picture still favors bilingualism protecting against symptoms of dementia. We suggest that the authors' recommendation that bilingualism be removed from consideration as a protective factor by policy makers is premature.

## Author contributions

All authors listed have made a substantial, direct and intellectual contribution to the work, and approved it for publication.

### Conflict of interest statement

The authors declare that the research was conducted in the absence of any commercial or financial relationships that could be construed as a potential conflict of interest.
